# Neuroprotective and Neuromodulatory Potential of *Valeriana officinalis*, *Passiflora incarnata*, and *Ginkgo biloba*: Efficacy, Safety, and Regulatory Aspects

**DOI:** 10.3390/plants15172694

**Published:** 2026-09-02

**Authors:** Lidia Mielczarek, Magda Frankowska, Anna Szurpnicka, Katarzyna Lubelska

**Affiliations:** 1 Department of Biochemistry and Biopharmaceutics, National Medicines Institute, Chelmska 30/34 Str., 00-725 Warsaw, Poland; 2 Department of Natural Products and Antibiotics, National Medicines Institute, Chelmska 30/34 Str., 00-725 Warsaw, Poland

**Keywords:** *Valeriana officinalis*, *Passiflora incarnata*, *Ginkgo biloba*, phytotherapy, CNS disorders, insomnia, anxiety

## Abstract

Valerian (*Valeriana officinalis* L.), passionflower (*Passiflora incarnata* L.), and ginkgo (*Ginkgo biloba* L.) are among the most widely used medicinal plants in European phytotherapy, with well-documented traditional use for anxiety, insomnia, cognitive decline, attention-deficit/hyperactivity disorder (ADHD), and Parkinson’s disease. This narrative review critically analyses available clinical, pharmacological, toxicological, and regulatory data on these three species in the context of central nervous system (CNS) disorders. A comprehensive literature search was conducted in PubMed, Scopus, and the Cochrane Library; regulatory documents from the European Medicines Agency (EMA), the European Scientific Cooperative on Phytotherapy (ESCOP), and the World Health Organization (WHO) were also included. Valerian showed the most consistent clinical evidence for an improvement in sleep quality and reduction in anxiety. Passionflower demonstrated anxiolytic and mild sedative effects in several controlled trials. Ginkgo showed clinically relevant benefits in mild cognitive impairment, dementia, and vertigo. All three plants exhibited acceptable safety profiles at the recommended doses; however, significant drug interactions were identified, notably ginkgo’s inhibition of CYP2C19 and antiplatelet effects, and valerian’s potentiation of CNS depressants. Regulatory frameworks across EU member states remain inconsistent. Further high-quality randomized controlled trials with standardized extract characterization are needed.

## 1. Introduction

Neurological and neuropsychiatric disorders (NDs), which include ADHD, Parkinson’s disease, migraine, insomnia, anxiety disorders, and dementia, are a varied group of conditions that involve the loss of neurons and worsening degeneration of various areas of the nervous system. To date, effective therapies to slow down, stop, or prevent neurodegenerative diseases have not been developed. Commonly used treatments are aimed at alleviating symptoms, not treating the cause of the disease [1]. Although medications are available, their efficacy is often limited and they may have severe or unacceptable adverse effects in patients. Due to the limitations of conventional therapies, both medical professionals and patients are increasingly seeking alternative, well-tolerated therapies. Medicinal plants and their preparations have gained increased interest—not only because of their use in traditional medicine, but also owing to growing preclinical and clinical data indicating their potential efficacy within the CNS [1,2]. In comparison to those for synthetic drugs, the frequency and nature of the adverse effects for herbal products are milder [3], although herbal preparations require longer use [4]. In contrast to synthetic chemical drugs, which usually contain a single active substance, have a known composition, and have specific therapeutic activity, herbal preparations contain extracts of varying composition. This variation results from species differences, climate conditions, geographical location, seasonal changes, and, most importantly, the extraction method. The active ingredients present in the extracts are typically identified; however, it is not always known which are active compounds and which are just adjuvants. Hence, dosing recommendations are usually based on the amount of the whole extract [5,6]. Information on the efficacy and safety of herbal preparations is often diffuse and equivocal and requires critical analysis. There are no complex reviews that contain not only the results of clinical trials but also the regulatory aspects, toxicology information, and limitations of use (e.g., interactions, contraindications, use in children and elderly). To address this literature gap, within this focused review, the authors describe the current and most reliable data on the use of *V. officinalis*, *P. incarnata*, and *G. biloba* in the context of chosen NDs, based on EBM data. Potential clinical benefits are included, while the risk of adverse effects is also described, and legal regulations surrounding the possibility of using the raw materials as dietary supplements are mentioned, along with safety assessments at the European level.

Valerian (*Valeriana officinalis*), passionflower or true passionflower (*Passiflora incarnata*), and ginkgo or maidenhair tree (*Ginkgo biloba*) have established statuses in European phytotherapy; they have EMA, ESCOP, and WHO monographs [7,8,9,10,11,12,13,14,15,16,17,18,19,20,21]. They are also important components of traditional medicine, which has recently been a source of inspiration for natural therapies. They are the source of raw plant materials used in therapy for neurodegenerative disorders, including anxiety disorders, insomnia, nervous distress, memory and concentration disturbances, and even ADHD and Parkinson’s disease.

## 2. Methods

This work is a narrative review. Its aim is to summarize and critically discuss the clinical, toxicological, and regulatory evidence regarding *Valeriana officinalis*, *Passiflora incarnata*, and *Ginkgo biloba* in disorders of the central nervous system, rather than to answer a single predefined question through a systematic protocol.

A literature search was carried out in three databases: PubMed, Scopus, and the Cochrane Library. The search covered the period from 1980 to 2025. For each species, the search combined the plant name, in both its common and scientific forms, with terms for the CNS indications of interest and for safety: insomnia, sleep, sedative, sleep-inducing, anxiety, anxiolytic, ADHD, dementia, parkinsonism, clinical trial, toxicity, safety, and adverse effects. The plant block and the indication-and-safety block were combined with the AND operator, and the terms within each block with OR. The searches identified 2281 records in PubMed, 8720 in Scopus, and 18 systematic reviews in the Cochrane Library, giving 11,019 records in total.

Regulatory and pharmacopeial documents were identified separately through a direct search of the relevant sources (EMA/HMPC, ESCOP, and WHO monographs; the European, British, and United States Pharmacopoeias; the EFSA compendium; and the national regulations of EU member states), and are therefore reported outside the database flow.

The inclusion criteria were clinical trials, systematic reviews, and meta-analyses reporting on the efficacy or safety of the three species in CNS-related conditions. A study was included when the effect could be attributed to one of the three species: for multi-ingredient preparations, only studies in which the contribution of the individual plant was identifiable were retained. Priority was given to full-text articles and to the most recent data. The exclusion criteria were studies on Valeriana or Passiflora species other than *V. officinalis* and *P. incarnata*; studies from which the effect of the plant of interest could not be separated; and records without accessible full texts.

**Figure 1:**
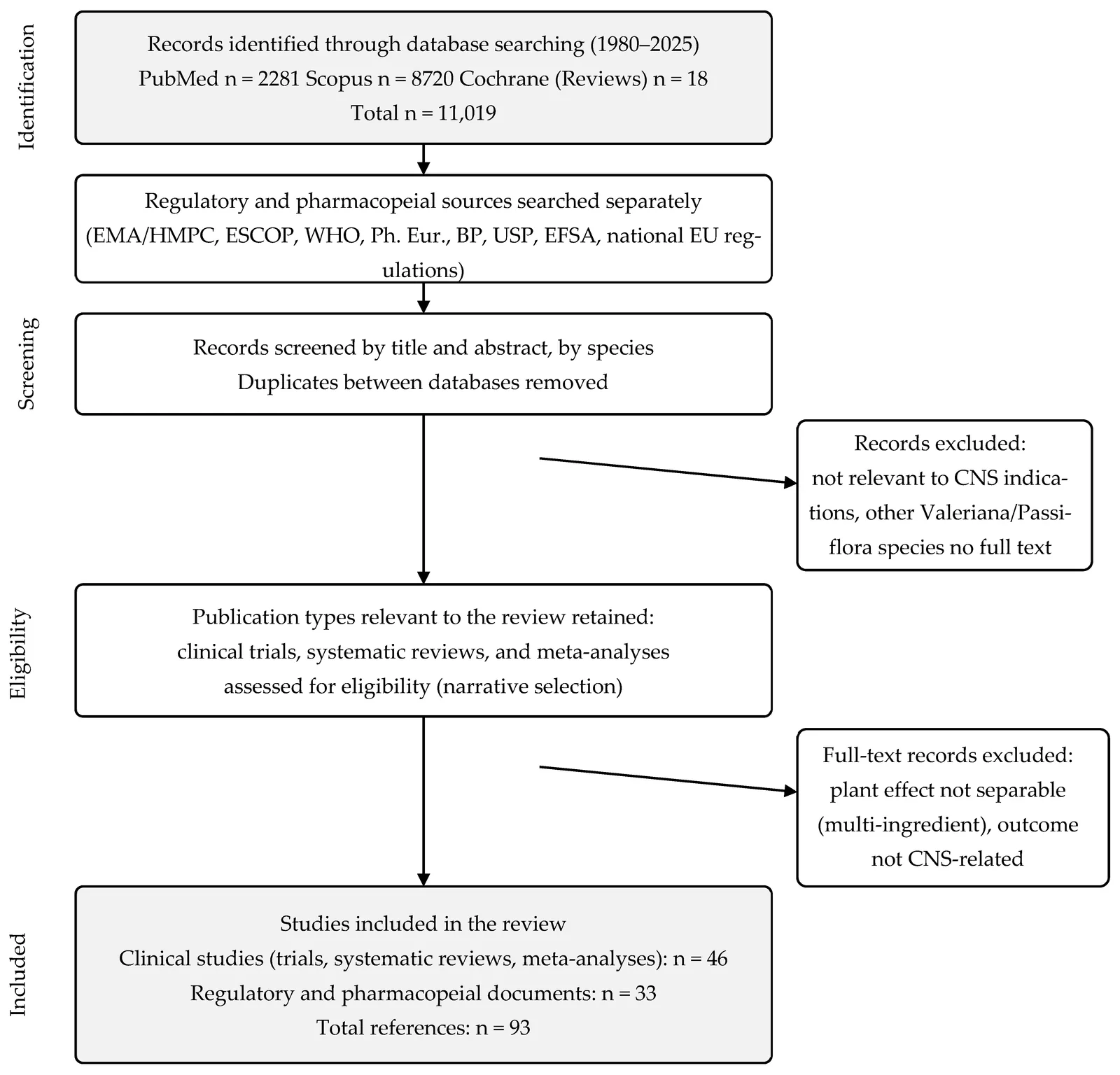
Narrative review flow diagram for the selection of studies on *Valeriana officinalis*, *Passiflora incarnata*, and *Ginkgo biloba*. As this is a narrative review, records were selected based on relevance rather than through a systematic screening of every retrieved record, and a formal risk-of-bias assessment was not performed.

Records were retrieved separately for each species and screened for relevance by title and abstract, and duplicates between databases were manually removed based on the DOI and title. Study selection was divided among the authors by species, and each included item was subsequently verified by the remaining authors. As this is a narrative review, records were selected based on relevance rather than through a systematic screening of every retrieved record, and a formal risk-of-bias assessment using a standardized instrument was not performed. Instead, the design, sample size, and main limitations of the key clinical trials are discussed in Section 5. The flow of records through the review is summarized in Figure 1.

## 3. Botanical Characteristics

*Valeriana officinalis* L. (*V. officinalis*) is a perennial of the Caprifoliaceae family, native to Europe, Asia, and North America [22]. The species has several varieties differing in morphology and chemical composition. Its main active constituents are ester iridoids known as valepotriates (valtrate, acetylvaltrate, dihydrovaltrate, and related compounds), present in the root and rhizome at about 1–2%. Because these esters carry different acids (e.g., hydroxyisovaleric, isocaproic, acetic), extracts of varying composition may occur. The detailed chemical composition is given in Table 1.

*Passiflora incarnata* L. (*P. incarnata*) belongs to the Passifloraceae family and is a perennial vine [23], originally from the southern United States and later introduced to South and Southeast Asia [24]. It is often confused with *Passiflora edulis*, which shares similar morphology but is grown for food and has no CNS activity. Chemical and chromatographic identity studies, most comprehensively a 2017 comparison of twelve *Passiflora* species, confirmed that the two are distinct [3,25]. The detailed chemical composition is given in Table 1.

*Ginkgo biloba* L. (*G. biloba*), of the Ginkgoaceae family, is native to south-eastern China and cultivated in East Asia, parts of Europe, and the United States [7,26]. A relic dioecious tree and the only living species of the order Ginkgoales, it is often called a living fossil and can reach an age of up to 2000 years [27].

## 4. Traditional Application

### 4.1. Valeriana officinalis

The root and rhizome of *V. officinalis* have been used in European herbal medicine for centuries as a sedative and sleep aid and in the treatment of anxiety, hysteria, and hypochondria, as well as neurodegenerative conditions such as Parkinson’s and Alzheimer’s disease. In traditional Chinese medicine, they are used for anxiety, insomnia, and manic depression [28].

### 4.2. Passiflora incarnata

The use of passionflower dates back to pre-Columbian North America, where Native American peoples used it as a sedative and sleep-inducing remedy. It was brought to Europe by Spanish colonizers and entered European herbal medicine for mental distress, anxiety, and mild sleep disturbance, as well as for indigestion and mild infections [3]. Across South America, Asia, Africa, and Australia, it is still used in folk medicine, mainly for its sedative, anxiolytic, antispasmodic, and analgesic effects, and in some regions for morphine or opioid withdrawal, neuralgia, and painful menstruation [3]. Today, it is a common phytotherapeutic mild sedative and anxiolytic.

### 4.3. Ginkgo biloba

Ginkgo has a long medicinal history, dating back to about 2800 BCE, with the internal use of the leaves first recorded in the Chinese materia medica *Ben Cao Pin Hui Jing Yao* (1505) [29]. The leaves were traditionally used for asthma, peripheral and coronary circulatory disorders, and other cardiovascular complaints, and modern Chinese pharmacopeias still list them for cardiac and pulmonary indications. In Europe, leaf preparations have been used only since the 1950s [30].

Roasted ginkgo seeds are a culinary item in China, Korea, and Japan [27], whereas fresh seeds are regarded as toxic by the Chinese Pharmacopoeia [31]. Poisonings, including fatalities, were reported in Japan, peaking during the food shortages of the 1940s and 1950s, with a case fatality of about 13% (22 deaths per 170 reported cases); no deaths have been recorded since 1969 [31].

## 5. Effects of *Valeriana officinalis*, *Passiflora incarnata*, and *Ginkgo biloba* on Alleviating Neurodegenerative Disorders: Dosages and Safety

Over the last few years, the number of clinical trials registered at ClinicalTrials.gov has grown, with some referring to organizations such as the EMA and WHO in their reports. Not all the study results have been published. The authors of this review focused on available trial results and summarize them below and in Table 2.

### 5.1. Effects of Valeriana officinalis and Passiflora incarnata on Insomnia

Insomnia is a sleep disorder that affects a growing number of adults worldwide, with an estimated 852 million people affected (global prevalence: 16.2%), and has become a serious public health problem [32]. The treatment of chronic insomnia often includes prescription medications, such as benzodiazepines and sleep-inducing medications, but they have many adverse effects, such as addiction, headaches, nightmares, tiredness during the day, nausea, disorientation, and loss of balance resulting in falls [33]. Other pharmacological therapeutic methods, such as anti-psychotics and antidepressants, also cause significant adverse effects. Nevertheless, they are commonly prescribed “beyond indications” in the case of chronic insomnia, especially in the elderly. Hence, alternative therapeutic methods for sleeping disorders are sought, prompting studies on the use of traditional herbs [2].

Research indicates that *Valeriana officinalis* and *Passiflora incarnata* have positive effects in insomnia. Donath et al. showed that *Valeriana officinalis* root extract improves the sleep structure and perception of sleep in people with insomnia, and hence, it can be recommended in the therapy of mild psycho-physiological insomnia [34]. A positive effect of valerian was also confirmed in a study involving children. Hintelmann (2002) [35] reported that, in a clinical trial (Table 2), the use of *V. officinalis* root extract in children with anxiety and/or difficulty falling asleep due to nervousness showed “good” or “very good” therapy efficiency in 89% of children who had anxiety, 100% of children with difficulties falling asleep, and 87% of children with both symptoms. Pre-existing simultaneously occurring symptoms, such as gastro-intestinal disorders, tiredness during the day, and weariness, were also alleviated in a high percentage of children. As this was an uncontrolled observational study, these figures are indicative rather than confirmatory [8,35].

In an experiment, Ngan et al. showed that brewed flowers of *P. incarnata* improved sleep comfort in healthy adults with mild swings in quality of sleep. Among six analyzed indicators according to a sleep diary, the quality of sleep proved to be significantly better when the passionflower infusion was administered, as compared to a placebo. The mean value (standard deviation) of the subjective parameter was 3.83 (0.61) versus 3.57 (0.56) [36]. Also, a randomized clinical double-blind and placebo-controlled study showed a positive effect of passionflower extract on objective parameters of sleep, including total sleeping time (TST), in adults with insomnia (passionflower vs. placebo, 23.05  ±  54.26 vs. −0.16  ±  53.12; *p*  =  0.049). Sleep efficiency and wake after sleep onset (WASO) significantly improved after 2 weeks in the Passionflower group, but there was no difference compared with the placebo group (Table 2) [37].

A recent study also reported reduced anxiety and improved sleep quality after *Passiflora incarnata* in children and adolescents, although the participants had feeding and eating disorders, which limits generalisation to the wider paediatric population [38].

Very valuable information on the potential use of both *V. officinalis* and *P. incarnata* in supporting sleep has been provided by studies comparing the effects of these herbs with those of substances used in conventional therapies. Studies by Ziegler et al. comparing the effects of valerian and oxazepam have shown that valerian extract at a dose of 600 mg/day had comparable efficacy to oxazepam at a dose of 10 mg/day in the therapy of inorganic insomnia (Table 2) [39].

It was shown that *Passiflora incarnata* also has an effect on sleeping disorders. In a clinical trial, diazepam, a placebo, and passionflower extract had similar influences on sleep quality in human subjects, causing similar decreases in mental alertness [3]. These results indicate the need for further research on these herbs, including comparative studies with existing medications, to find effective and safe alternative therapies. The literature indicates minor side effects of these herbs.

In a review article, Taibi et al. described that ethanol extracts of valerian roots had adverse effects including headaches; morning “hangovers”; gastro-intestinal problems, such as diarrhea; drowsiness; intellectual disability; depression; irritability; dizziness; and nausea [40]. According to Leathwood et al., the symptoms reported for aqueous extracts included headaches, significantly increased drowsiness in the morning (at a dose of 900 mg, as compared to 450 mg), and nausea [41]. Although these results have not been reported in current studies, they were used by the EMA in its assessment report on *Valeriana officinalis* L., radix and *Valeriana officinalis* L., aetheroleum [8]. At the same time, the risk of interactions should be considered when using herbs and medicinal products simultaneously. There is a described case of adverse effects in a patient who took pills containing a dry extract from *Valeriana officinalis* vines and root and the aboveground parts of *Passiflora incarnata* while being treated with lorazepam (no information about the doses and standardization). The patient reported trembling of hands, dizziness, pulsation, and tiredness of muscles 32 h before the clinical diagnosis. Upon medical interview, generalized anxiety syndrome and sleeping disorders were diagnosed. It was concluded that the symptoms most probably occurred due to an interaction between the herbal medication and lorazepam [42].

### 5.2. Effects of Valeriana officinalis, Passiflora incarnata, and Ginkgo biloba on Alleviating Anxiety and Pain of Neurological Origin

The treatment of generalized anxiety disorders (GADs) is similar to insomnia therapy: patients can use medications such as benzodiazepines, antidepressants, and pregabalin (an anti-seizure drug), but there are also effective psychological therapies, such as behavioral therapy, training in relaxation reactions, training in meditation and awareness, and cognitive behavioral therapy, which is the best-studied and most commonly used [43]. Similarly, cognitive behavioral therapies have shown efficacy in migraine treatment. The biological basis of migraine is not fully known yet; it comprises a complex interaction of environmental, biochemical, epigenetic, and genetic factors. A still-growing number of patients use herbal and plant therapies to treat migraine, and increasing clinical and preclinical evidence supports their efficacy [44,45]. Below, a summary of the clinical studies described in Table 2 related to therapy aimed at alleviating anxiety and pain of neurological origin is presented.

In a randomized double-blind clinical trial, the efficacy of *Passiflora incarnata* extract was comparable to that of oxazepam therapy in the treatment of GAD. According to Akhondzadeh et al., this offers the possibility to develop a replacement therapy in patients with generalized anxiety disorder (Table 2) [3,45]. The potential of passionflower extract as a replacement therapy was confirmed by a clinical study, in which Movafegh et al. administered a single 500 mg tablet of passionflower extract to volunteers 90 min before a procedure, which reduced preoperative anxiety without the need for additional sedatives [46].

These results were also confirmed by Aslanargun et al. (2012)’s prospective, randomized, double-blind placebo-controlled study, which showed that taking syrup with *P. incarnata* decreased the clinical symptoms of anxiety before lumbar anesthesia, without affecting psychomotor functions, the level of sedation, or hemodynamic parameters, and without causing adverse effects [47].

Another study showed that *P. incarnata* can complement existing therapies. Akhondzadeh et al. [48] showed that a faster reduction in mental disorders was observed for a group of patients treated for two weeks with clonidine and *Passiflora incarnata* extract (the reduction had already occurred on the second day, but not in the clonidine group), as compared to patients treated with clonidine and a placebo. Statistical analysis of data on mental and physical symptoms showed that therapy comprising *Passiflora incarnata* and clonidine decreased the psychical symptoms (e.g., insomnia, problems with sleep, dysphoria, anxiety, agitation, irritability, and substance craving) to a greater degree than physical symptoms after the discontinuation of opioids, as compared to patients treated with clonidine only [48].

A positive effect during medical intervention was also observed for *Valeriana officinalis*. Pinheiro et al. reported that subjects taking a product containing *V. officinalis* were calmer and more relaxed (85% of patients) compared to subjects taking a placebo (45% of patients) during oral surgery. The study showed that the product with *V. officinalis* had a greater effect (as compared to placebo) in terms of the maintenance of blood pressure and pulse after the procedure, and hence, it was more effective in reducing anxiety. Only mild adverse effects were noted [49].

### 5.3. Effects of Valeriana officinalis, Passiflora incarnata, and Ginkgo biloba on Chosen Neurodegenerative Conditions, i.e., Parkinson’s Disease, Alzheimer’s Disease, Dementia, and ADHD

The WHO’s report shows that neurological conditions now affect more than 40% of the global population—over 3 billion people [50].

To date, no effective therapies have been developed to slow, stop, or prevent neurodegenerative diseases. However, research indicates that herbal therapies may have the potential to alleviate symptoms while minimizing side effects and may also complement conventional therapies [1].

An observational study was conducted in which children with hyperactivity and problems with concentration but not fulfilling ADHD criteria were administered a daily dose of a product with the brand name Sandrin, containing 640 mg of ethanol extract from *Valeriana officinalis* root and 320 mg of lemon balm extract. A decrease in the percentage of children with strong/very strong symptoms of poor ability to concentrate and with symptoms of hyperactivity and impulsiveness was observed. Problems concerning social behaviors and sleep, and the burden due to symptoms of hyperactivity and concentration were significantly decreased in parents’ assessment. Only two children exhibited mild, transient adverse effects (Table 2) [51].

*Valeriana officinalis* also has potential application in the treatment of obsessive–compulsive disorders (OCDs), which are common neuropsychiatric conditions, as shown in an 8-week pilot randomized double-blind study. The results showed beneficial effects of *Valeriana officinalis* extract as compared to a placebo [52].

A randomized, double-blind clinical study evaluating the effects of *P. incarnata* extract in ADHD therapy demonstrated the health-promoting effects of this herb with fewer side effects. The study showed that the extract had the same therapeutic effect as methylphenidate. In subjects in the methylphenidate group, adverse effects occurred more often than in the *Passiflora incarnata* group (the following effects were observed: decreased appetite, anxiety, nervousness, decreased body weight, headache, and difficulty falling asleep). These results suggest that the main benefit of using *Passiflora incarnata* in ADHD therapy (apart from efficacy comparable to that of methylphenidate) is a decrease in adverse effects (Table 2). The absence of a significant difference from methylphenidate should be interpreted with caution, as the small sample size limits the power to detect non-inferiority [3].

Despite rare and mild side effects, science is still testing its full effectiveness. A few cases of reported adverse effects after the consumption of passionflower products were noted. A 34-year-old woman had severe nausea, extended vomiting, weakness, drowsiness, bradycardia, and ventricular rhythm disturbances, including ventricular bigeminy and ventricular tachycardia, after using a plant medicine at therapeutic doses, without medical supervision for 2 days. According to the manufacturer’s declaration, 1 tablet contained passionflower extract corresponding to 500 mg of active ingredients (other more detailed information is missing). The recommended dose was 0.5–1.0 g 3× a day (no other dosing information). A marked temporal relationship between using the plant product and the onset of the adverse effects was confirmed. Moreover, no cardiovascular or gastro-intestinal co-morbidities were found and there were no other causes of these symptoms. After discontinuation of the medication, the patient’s health improved. The authors of the article attribute the observed effects to harman alkaloids. These alkaloids, apart from their anti-stress effects [53], may cause bradycardia and ventricular disturbances of heart rhythm. They also affect neurotransmitter metabolism and may inhibit the action of intestinal smooth muscles [54].

According to literature data, large doses of *Passiflora incarnata* raw material (above 8–10 g) cause intensive headache and sometimes vision disturbances. Passionflower preparations may also intensify the effects of sedatives and ethanol [55].

A health-promoting effect regarding the relief of symptoms of neurodegenerative diseases was also demonstrated for ginkgo biloba; however, the effect was not comparable to that of conventionally used drugs and largely depended on the duration of use and the health condition of the study participants. A randomized, double-blind parallel-group study assessing the potential use of ginkgo products in ADHD treatment showed that significant differences were observed between the two groups on the Teacher ADHD rating scale and the Parent-IV rating scale, but the administration of *G. biloba* did not show efficacy comparable to that of methylphenidate [56].

Gessner et al.’s study in volunteers with age-related worsening of mental condition showed that, in terms of alertness, no significant efficacy of EGb 761 ginkgo extract was found compared to nicergoline and placebo (Table 2). Nevertheless, it was shown that the alertness of subjects with less favorable baseline situations could be markedly improved by the long-term intake of EGb 761 extract, as compared to nicergoline or placebo. According to the authors, the obtained results indicate that the long-term use of EGb 761 extract has positive effects in elderly people diagnosed with decreased mental abilities and alertness, and these effects are reflected at the behavioral level. In healthy people in good baseline conditions, almost no improvement was observed [57]. Even if not exactly the same, similar findings have been shown in a double-blind, placebo-controlled parallel-group study aimed at evaluating the effect of EGb761 ginkgo extract on cognitive function and quality of life in people with very mild disturbances in cognitive functions. The cognitive effects were more marked and consistent in subjects with worse memory function at the beginning of the study, as compared to subjects from the placebo group (Table 2) [58]. Herrschaft et al.’s multi-center, randomized, double-blind placebo-controlled study in patients with mild to moderate dementia (Alzheimer’s disease or vascular dementia) showed that neuropsychiatric functions were improved in subjects administered EGb 761 extract, while the improvement was nonsignificant in the patients in the placebo group. According to the authors, the use of EGb 761 extract in the case of mild and moderate dementia at a daily dose of 240 mg was safe and resulted in marked and clinically significant improvements in cognitive functions, functional parameters, and subjects’ quality of life (Table 2) [59]. These results are confirmed by the research of Cui et al., whose multi-center, randomized, open-label study conducted across 7 centers in China showed that, over 24 weeks of use, the EGb 761 extract improved the general cognitive fitness in subjects with mild to moderate ischemic brain stroke (Table 2) [60]. Another double-blind placebo-controlled study by Elsabagh et al. compared the effect of ginkgo extract LI 1370 on concentration, memory, and cognitive functions, showing that a single dose of LI 1370 extract significantly improved the efficacy in a task requiring long concentration and in a memory task of pattern recognition. However, no effects on working memory, planning, mental elasticity, and mood were observed. After 6 weeks of administration of the extract, no significant effects of the LI 1370 extract on mood or the results of cognitive tests were found, which, according to the study’s authors, indicates that young, healthy people develop tolerance to the extract’s effect [61].

A randomized, double-blind placebo-controlled clinical study reported by Mohebbi et al. in parallel groups showed that the studied ginkgo extract EGb may alleviate the severity of Parkinson’s disease symptoms and motor symptoms (the intensity of rest tremor (F = 23.904, *p* < 0.0001), the severity of motor symptoms (F = 24.126, *p* < 0.0001), rigidity (F = 4.487, *p* < 0.0001), and bradykinesia (F = 23.565, *p* < 0.0001)) and may result in positive changes in working memory (F = 4.643, *p* = 0.036) and recent memory (F = 8.258, *p* = 0.006). According to the authors, ginkgo extract can be used as an effective and safe agent to treat DIP in both patients diagnosed with psychotic disorders and those with mood disorders (Table 2) [62].

Zheng et al. also proved that ginkgo leaf extract (GBE, no information about standardization) yielded results comparable to those for donepezil in terms of improved physical and mental condition in subjects with Alzheimer’s disease (AD) (Table 2) [63]. In a phase 4 randomized double-blind placebo-controlled exploratory study, GBE extract improved the time and spatial parameters of walking in patients with mild cognitive impairment (MCI) in two-task conditions [64].

Vellas et al. reported different results. In the GuidAge trial, long-term use of a standardized EGb 761 extract did not reduce the risk of progression to Alzheimer’s disease compared with placebo. The study assessed whether long-term intake of EGb 761 could lower the incidence of Alzheimer’s disease in elderly people with memory complaints. After five years, possible Alzheimer’s disease was diagnosed in 61 subjects in the extract group. The frequency of adverse events was similar in both groups: 76 deaths in the extract group versus 82 in the placebo group, and 65 strokes versus 60, respectively, with no difference between groups in other hemorrhagic or cardiovascular events. In contrast to the smaller positive trials, this large, long-term randomized controlled trial found no preventive effect, which carries substantial weight given its design and duration [65].

**Table 2:** Summary of the key findings from clinical studies on *Valeriana officinalis*, *Passiflora incarnata*, and *Ginkgo biloba*.

Studied Plant	Extract	Form/Dose	Type of Study	Population Characteristics	Time of Use	Condition	Effect	Adverse Effects	Ref.
*V. officinalis,* root	Aqueous extract (DER 3:1)	Capsules 400 mg	Double-blind placebo-controlled study	10 healthy male subjects, aged 29 ± 3 years	4 nights	Quality of sleep	Shortening of sleep latency, improvement in quality of sleep	None	[8,41]
*V. officinalis,* root	Aqueous extract (DER 3:1)	Capsules 900 mg	Double-blind placebo-controlled study	8 subjects (4 female, 4 male), aged 21–26	5 nights	Quality of sleep	Estimated sleep latency and wake time after sleep onset were reduced by more than 50%	None	[8,41]
*V. officinalis,* root	Ethanol extract (DER 3–7:1)	Capsules 300 mg or 600 mg	Double-blind placebo-controlled, cross-over study	16 subjects (11 female, 5 male), aged 50–64	One dose	Sleeping disorders	Tendency for increased sleepiness with higher dose	None	[66]
*V. officinalis,* root	Ethanol extract (DER 3–7:1)	Tablets 600 mg/day	Double-blind placebo-controlled study, cross-over study	16 subjects (12 female, 4 male), aged 22–55, primary insomnia	2 weeks	Insomnia	Improved efficiency of sleep, increased slow-wave sleep; slow-wave sleep latency decreased from 21.3 min to 13.5 min. The percentage of slow-wave sleep time increased from 8.1% to 9.8%.	Hives, migraine, gastro-intestinal disorders	[34]
*V. officinalis,* root	Ethanol extract (DER 3–6:1)	Tablets 600 mg/day	Randomized, double-blind, multi-center study	186 subjects, aged 18–73, inorganic insomnia	6 weeks	Insomnia	Efficiency comparable to that of oxazepam (*p* < 0.01)	Mild to moderate; occurred in 28, 4 patients receiving valerian extract	[39]
*V. officinalis,* root	Aqueous ethanol extract (DER 3-5:1)	Syrup 600 mg/day, which according to the authors corresponded to 1.8–3.6 g of herbal substance	Pharmacological monitoring	130 children, aged 6–12, difficulty falling asleep, and anxiety	2–4 weeks	Difficulty with falling asleep, anxiety	Improvement in quality of sleep, decreased symptoms of nervousness After 4 weeks, the effectiveness of the therapy was “good” or “very good” in 89% of children who only experienced anxiety, 100% of children with difficulty falling asleep, and 87% of children with both symptoms.	None; no information	[8,35]
*V. officinalis,* root	No data on standardization and form	100 mg	Assessment of sedative properties	20 subjects (12 female, 8 male), aged 17–31	Single dose, 1 h before a surgical procedure	Assessment of sedative properties during oral surgery	Maintenance of blood pressure and pulse after the procedure -> effective in lowering anxiety	Sleepiness, relaxation of muscles—similar to placebo	[49]
*V. officinalis*	Aqueous extract from valerian root (DER 7:1:1, no data on extract standardization)	Capsules containing 765 mg/day	Pilot randomized double-blind study	31 adult patients (15 women and 16 men aged 18 to 60)	8 weeks	Treatment of obsessive–compulsive disorders	At the end of the study, there was a difference between the extract and placebo (no other information). According to the authors, *Valeriana officinalis* has potential application in treatment of OCD	Sleepiness, headaches, nausea, dry mouth, blurry vision, and constipations	[52]
*V. officinalis,* root	Sandrin preparation (no information about extract standardization)	640 mg of ethanol extract from valerian root WS 1014 (DER 3-6:1) and 320 mg of lemon balm extract WS 1303 (DER 4-6:1)	Observation study	169 children (1/3 of the subjects were girls and 2/3 were boys aged 6 to 11)	7 weeks	Therapy for children with hyperactivity and concentration disorders, but not fulfilling ADHD criteria	Percentage of children with strong/very strong symptoms: poor concentration ability decreased from 75% to 14%: - With hyperactivity symptoms: from 61% to 13%; - With impulsiveness: from 59% to 22%. Social behaviors, sleep, and burden due to symptoms of hyperactivity and concentration problems were significantly decreased	Mild, transient adverse effects in 2 children: fatigue, irritability, eye blinking	[51]
*V. officinalis, root*	70% ethanol root extract	600 mg (DER 5:1, no data about standardization); according to a monograph, this corresponds to 3 g of valerian root	Randomized, double-blind, placebo-controlled study	61 subjects (mean age 47 years, no information about gender) + 60 subjects, placebo group	Every day for 28 days	Insomnia	Improvement in quality of sleep, a feeling of being rested after sleeping and general well-being	A sensation of being dazed in the morning	[17]
*P. incarnata,* herb	Extract (DER: 5.9:1)	Capsules 1200 mg	Randomized, double-blind, placebo-controlled study	12 female subjects, aged 53.7 ± 5.6 years	Single dose	Quality of sleep	No effect on sleep EEG, similar loss of mental alertness to diazepam and placebo	Fatigue	[3]
*P. incarnata,* leaves, stems, seeds, flowers	Infusion	Infusion 2 g/250 mL) 3× daily	Double-blind placebo-controlled study	41 subjects (14 male, 27 female), aged 18–35	1 week	Quality of sleep	Improvement in quality of sleep, confirmed using polysomnography (PSG)	None	[36]
*P. incarnata,* leaves and fruit	Ethanol extract (8:2), standardized to vitexin (no data on its content in the extract)	Capsules 60 mg	Randomized, double-blind, placebo-controlled study	110 subjects (56.3% female), aged 18–59, insomnia	2 weeks	Insomnia	Increase in total time of sleep by 23.05 ± 54.26 min in the passiflora group	None	[37]
*P. incarnata,* herb	Liquid extract	Drops, 45 drops containing 11–15 mg of flavonoids as calculated to hyperoside in 100 mL of brew	Randomized, double-blind, placebo-controlled study	36 subjects, aged 19–47	4 weeks	Generalized anxiety	Efficiency comparable to that of oxazepam. In the oxazepam group, but not the Passiflora group, significant effects were observed by day 4. Post hoc testing revealed a significant reduction from baseline from day 4 in the oxazepam group and from day 7 in the passiflora group.	Nausea, drowsiness, disorientation	[3,67]
*P. incarnata*, herb	extract	Tablets 500 mg containing 1.01 mg benzoflavone	Randomized, double-blind, placebo-controlled study	60 subjects, aged 25–45	Single dose (90 min before the procedure)	Presurgical anxiety	Reduction in anxiety before surgery. There was a significant difference in the mean NRS anxiety scores measured over time between the control and passiflora groups	None	[46]
*P. incarnata,* herb	Aqueous extract	Syrup (700 mg/5 mL)	Randomized, double-blind, placebo-controlled study	60 subjects, aged 25–55	Single dose	Anxiety before anesthesia	Reduction in anxiety Baseline State Anxiety Inventory score obtained just before spinal anesthesia (STAI-S_1_) and Baseline Trait Anxiety Inventory (STAI-T_1_) scores were similar in both passiflora and control groups (*p* = 0.728 for STAI-S 1 and *p* = 0.141 for STAI-T 1).	None	[47]
*P. incarnata,* herb	Ethanol extract (5–7:1, 50%)	425 mg 3×/daily	Multi-center, prospective, non-intervention study	n = 153 women 75.2%, men 24.8%, aged below 95	12 weeks	Diagnosed nervous anxiety	Tolerance was assessed as very good or good in more than 99% of patients	Three cases of fatigue	[68]
*P. incarnata,* leaves and fruit of passionflower	Extract (60% ethanol, 8:2)	60 mg of extract every night for 2 weeks	Randomized double-blind study with placebo	45 subjects aged 18–59 (no information about gender)	2 weeks	Insomnia	Extension of the total sleep time in the study group; improvement in sleep efficiency, waking up after falling asleep, improvement in scores for depression, anxiety, and stress, as compared to baseline	None	[68]
*P. incarnata*	Extract (no information about the strength of the extract)	0.8 mg clonidine + 60 drops of liquid extract from passionflower	No information about the type of study	65 male volunteers, aged 28–44	2 weeks	Support for clonidine therapy, after discontinuation of opiates	Decrease in psychical symptoms, e.g., insomnia, sleep disorders, dysphoria, anxiety, agitation, irritability, and substance craving, as compared to the control group. The means that the total score for the clonidine group was significantly higher than that for the passiflora group on day 14 (t à 2·716, d.f. à 28, P à 0·011).	Not given	[48]
*P. incarnata*	Passionflower extract (no information about the strength of the extract)	Tablets 0.04 mg/kg b.w./day in divided dose	Randomized double-blind clinical trial	34 school children (23 boys and 11 girls)	8 weeks	Assessment of efficiency of *P. incarnata* in people with ADHD	No significant differences in effects between the two study groups were observed at day 0 (baseline) on the Parent ADHD Rating Scale (t = 0.05; df = 32; *p* = 0.95). Both groups showed a significant improvement over the 8 weeks of treatment (Greenhouse–Geisser correction; F = 46.51, df = 2.46, *p* < 0.000) and the trend was linear.	Group treated with methylphenidate: reduced appetite, anxiety, nervousness, decrease in body weight, headache, and difficulties with falling asleep, more often than in the group that used *Passiflora incarnata*.	[3]
*Ginkgo biloba*, leaves	Extract EGb 761	Tablets 240 mg/day	Multi-center, randomized, double-blind placebo-controlled study	402 subjects aged >50, mild to moderate dementia	24 weeks	Alzheimer’s disease, vascular dementia	Significant improvement in cognitive functions and quality of life. Improvement of 2.2 ± 3.5 points on the SKT in the EGb 761 group and 0.3 ± 3.7 points in placebo; NPI score improved by 4.6 ± 7.1 in the EGb 761 group and by 2.1 ± 6.5 in the placebo Group; both drug–placebo comparisons were significant at *p* < 0.001	In 44.4% (headache, dizziness, upper respiratory tract infection, insomnia, hypertension)	[59]
*Ginkgo biloba*	Extract EGb 761	Tablets 2 × 120 mg/day	Randomized, double-blind, placebo-controlled study	2854 subjects aged ≥ 70 with memory problems	5 years	Prevention of Alzheimer’s disease	No decrease in the risk of Alzheimer’s disease	Similar frequency of adverse effects in both groups	[65]
*Ginkgo biloba*	Extract GBE	Tablets 3 × 150 mg/day	Randomized, double-blind, placebo-controlled study	150 subjects with Alzheimer’s disease (aged 50–85)	6 months	Alzheimer’s disease	Effect similar to that of donepezil; no advantage of the combined therapy. In GBE group, modified Hachinski score, Hamilton anxiety scale, and Clinical Dementia Rating mean (CD) were, respectively, 0.96 (0.81), 2.48 (2.60), and 1.08 (0.27); in the doneprazil group, 0.78 (0.71), 1.72 (2.12), and 1.24 (0.43); in the combined group, 0.86 (0.83), 2.00 (1.99), and 1.22 (0.42)	No information about adverse effects	[63]
*Ginkgo biloba*	Extract EGb	Tablets 3 × 80 mg/day	Randomized, double-blind, placebo-controlled study	63 subjects with drug-induced parkinsonism (aged 49–52)	3 months	Drug-induced parkinsonism (DIP)	DIP motor symptoms were reduced from 17.97 to 11.83 in the *Ginkgo biloba* group. The rate of change in the severity of the tremor decreased from 9.56 to 6.03 in the *Ginkgo biloba* group. The rate of change in the severity of bradykinesia symptoms decreased from 5.5 to 3.67 in the group receiving *Ginkgo biloba*. Working increased from 3.66 to 4.21 in the group receiving *Ginkgo biloba*.	1 severe rash, mild stomach problems, headache, and dizziness	[62]
*Ginkgo biloba*	Extract LI 1370	Tablets 2 × 120 mg/day	Randomized, double-blind, placebo-controlled, exploratory study	50 subjects with MCI (aged 50–85) and walking disturbances	6 months + 6 months open phase	MCI and walking disturbances	Improvement in walking parameters in two-task conditions; dual-task-related cadence increased in the intervention compared to the control group (*p* = 0.019, d = 0.71). No significant changes, but GBE-associated numerical nonsignificant trends were found after 6-month treatment for dual-task-related gait velocity and stride time variability.	No differences in frequency of adverse effects, effects not related to the preparation	[64]
*Ginkgo biloba*, leaves	Extract (no information about standardization)	Tablets (Ginko T.D.) 80–120 mg/day	Randomized, double-blind, placebo-controlled study	50 children with ADHD (39 boys, 11 girls, aged 6–14)	6 weeks	ADHD	Lower efficiency than methylphenidate. For the Parent ADHD Rating Scale, the changes at the endpoint compared to baseline were −6.52 ± 11.43 and −15.92 ± 11.44 for Ginko and methyphenidate, respectively. For the Teacher ADHD Rating Scale, the changes at the endpoint compared to baseline were −0.84 ± 6.79 and −14.04 ± 8.67 for Ginko and methyphenidate, respectively	Decreased appetite, headache, insomnia (more frequently in methylphenidate group)	[56]
*Ginkgolide B* isolated from leaves and bark of *Ginkgo biloba*	Complex of ginkgolide B/co-enzyme Q10/riboflavin/magnesium	Lack of detailed quantitative composition	Pilot study	119 subjects (boys and girls, mean age 9.7 ± 1.42 years)	2 × daily for 3 months	Prevention of primary headache, i.e., migraine without aura	Average frequency of migraine in a month was significantly decreased, from 9.71 to 4.53 attacks	No information	[50]
*Ginkgolide B* isolated from leaves and bark of *Ginkgo biloba*	Complex of ginkgolide B/co-enzyme Q10/vitamin B2/magnesium	80 mg ginkgolide B, 20 mg co-enzyme Q10, 1.6 mg vitamin B2, and 300 mg magnesium	Pilot open-label study	30 subjects aged 8 to 18 (boys and girls, mean age 13.5 ± 2.2 years)	2 × daily for 3 months	Prevention of primary headache, i.e., migraine without aura	The number of migraine attacks in a month decreased after 3 months of product use, as compared to the baseline value before the study. After 1 year of observation, average number of headache days in a month decreased significantly from about 7.2 to 1.6.	No information	[69]
*Ginkgo biloba*	Dried extract EGb 761	3 × 40 mg/day of extract EGb 761	12 weeks randomized, double-blind, placebo- controlled, parallel	60 volunteers (aged 57–77, no information about gender)	12 weeks	Age-related, worsening of mental condition	Long-term use of extract Egb 761 has positive effect on elderly people diagnosed with decreased mental abilities and alertness, and this effect is reflected at the behavioral level. In healthy people in good baseline condition, almost no improvement was observed.	No information	[57]
*Ginkgo biloba*, leaves	Ginkgo leaf extract LI 1370 (standardized to 25% of flavone glycosides and 6% of lactone terpenes)	120 mg/day once or for 6 weeks	Double-blind placebo-controlled clinical trial	92 healthy volunteers (aged 18–26, men and women)	One time or continuously over 6 weeks; 52 participants once 120 mg/day of extract (n = 26) or placebo (n = 26); 40 subjects 6 weeks extract LI 1370 at a dose of 120 mg/day (n = 20) or placebo (n = 20)	Influence on concentration, memory, and mental functions in healthy volunteers	In the sustained attention test, there was a significant treatment × speed interaction on the PASAT (F1,50 = 7.1, *p* < 0.02), as the ginkgo group outperformed the placebo group as the task became more difficult. There was a significant effect at the most difficult stage of the task (t50 = 2.1, *p* < 0.04), as the ginkgo group obtained significantly more correct responses than the placebo group. No significant Week × Treatment interactions on any of the tests of episodic memory. There was, however, a significant effect of week for the latency to respond on the PRM task (F1,38 = 11.8, *p* < 0.002) and the SRM task (F1,38 = 5.6, *p* < 0.03) and on the number of recalled words (F1,38 = 31.2, *p* < 0.0001) and pictures (F1,38 = 4.9, *p* < 0.04). No effects on working memory, planning, mental flexibility, or mood	No information	[61]
*Ginkgo biloba*, leaves	Dry extract from ginkgo biloba (100 mg contained 24% flavonoids and 6.1% terpenoids (2.7% bilobalide, 1.7% ginkgolide A, 0.9% ginkgolide B and 0.8% ginkgolide C)	80 mg/day	Double-blind placebo-controlled study	48 healthy male subjects (aged 60–70)	8 months	Tests of cognitive changes	The experimental group showed a decrease in blood viscosity, improved cerebral perfusion in certain regions, and improvements in general cognitive functioning	No information	[70]
*Ginkgo biloba*	*Ginkgo biloba* extract EGb 761	240 mg/day of EGb 761 extract	Multi-center randomized open-label study in 7 centers in China	201 subjects of both sexes, aged ≥50, where 100 were assigned to the group taking EGb 761 extract, and 101 subjects were assigned to the reference group; the included subjects obtained 20 or fewer points on the stroke scale of the National Institute of Health (NIHSS), over a period of 7 to 14 days after the stroke	24 weeks	Treatment of cognitive dysfunctions, functional disturbances, neuropsychiatric symptoms, and dementia in subjects after an acute ischemic brain stroke	Over 24 weeks of use, the Egb 761 extract improved the general cognitive fitness in subjects with mild to moderate ischemic brain stroke	3% of subjects (nasopharyngitis) in both groups	[60]
*Ginkgo biloba*	*Ginkgo biloba* extract EGb 761	240 mg/1× daily of EGb 761 extract or placebo	Double-blind, placebo-controlled study in parallel groups	300 subjects (aged 45–65, women and men)	12 weeks, 1× daily	Effect on cognitive functions and quality of life of people with very mild cognitive function disturbances	*Ginkgo biloba* extract EGb 761 improved cognitive functioning and aspects of quality of life of the subjects with very mild disturbances of cognitive functions. The subjects also showed significant improvement in the concentration test (Vienna Test System-Work Performance Series), memory test (Wechsler’s Memory Scale III), and perceived physical health (score of Quality of Life Questionnaire SF-36)	Gastro-intestinal symptoms and infections and parasitic infestations	[58]

### 5.4. Overall Strength of Evidence

The clinical evidence differs markedly across the three species and across indications. For valerian and passionflower in insomnia and anxiety, several randomized, placebo-controlled or active-comparator trials are available, although most enrolled small samples (typically fewer than 100 participants); used short treatment periods; and, in many cases, did not report extract standardization. The direction of effect is reasonably consistent, which supports a moderate level of evidence for a mild hypnotic and anxiolytic effect, while precise effect sizes remain uncertain.

For ginkgo in mild cognitive impairment and dementia, the evidence base is larger and includes multi-center randomized trials with standardized extracts (mainly EGb 761), which places this indication on the firmest footing of all those reviewed. It is counterbalanced, however, by the large GuidAge trial, which found no preventive effect on progression to Alzheimer’s disease (Table 3).

For the remaining indications, namely ADHD, Parkinson’s disease, and migraine, the evidence is preliminary. It rests on single trials, small or uncontrolled studies, or fixed-combination products from which the effect of the individual plant cannot be isolated. These results are hypothesis-generating and require confirmation in adequately powered randomized controlled trials before any clinical recommendation can be made.

## 6. Toxicity

Experimental data on the toxicological properties of *Valeriana officinalis*, *Passiflora incarnata*, and *Ginkgo biloba* are limited.

No studies on the carcinogenicity and reproductive toxicity of valerian root and *Passiflora incarnata* and their preparations are available (European Medicines Agency, 2016a) [9]. Studies on *G. biloba* extracts showed no effects that were mutagenic, carcinogenic, or toxic to reproduction [7].

### 6.1. Valeriana officinalis

For native extracts of *Valeriana officinalis* or compounds isolated from them, different experiments have determined low toxicity, with the exception of valepotriates and products of their decomposition. Valepotriates, present mainly in freshly collected roots, are very short-lived due to their unstable epoxy structure and they decompose in various conditions (bases, acids, storage) to baldrinals and their derivatives. As valepotriates degrade rapidly upon storage and their oral bioavailability seems very low, the in vitro and in vivo effects observed for isolated valepotriates and their decomposition products or valepotriate-containing extracts should be disregarded when assessing valerian roots, according to EMA. Alkylating, cytotoxic, and mutagenic effects of valepotriates and their decomposition products were described; however, the compounds are not detectable in valerian root extracts or they are present in very low quantities [8]. The WHO also stated that they do not pose a threat, despite concerns related to valepotriates’ cytotoxicity shown in vitro and alkylating effect in vitro.

### 6.2. Passiflora incarnata

No cases of passionflower extract overdose in people have been reported. The American Food and Drug Agency (FDA) classified it as Generally Regarded as Safe (GRAS) [3].

### 6.3. Ginkgo biloba

The EMA’s report states that ginkgo leaf extract (no data on standardization) was positive for genetic mutations in bacteria and yielded an unequivocal and negative result for chromosome mutations [10,29].

Native extracts of ginkgo contain a group of alkylphenols (e.g., ginkgolic acids, ginkgole, bilobole) showing potential allergic effects on contact and toxic effects [11,29]. According to the EMA, the acceptable maximum concentration of the sum of these compounds is 5 mg/kg [29].

Ginkgo fresh seeds (GBSs) contain numerous allergenic and toxic substances that may cause health problems, which limits their intake. GBSs contain ginkgotoxin, a vitamin B6 analog that causes poisoning, tonic–clonic seizures, vomiting, and neurotoxic changes by lowering γ-aminobutyric acid (GABA) levels in the brain. The concentration of ginkgotoxin in fresh GBSs ranges from 170 to 404 mg/kg, and toxicological reports have indicated that concentrations above 170 mg/kg cause poisoning [12]. The 5′-glycoside of ginkgotoxin shows similar activity. Ginkgolic acid present in fresh seeds shows cytotoxic, mutagenic, genotoxic, neurotoxic, and allergenic effects. Also, glycoproteins present in GBSs can be allergenic; clinically, allergy may cause diarrhea, seizure, dilation of pupils, nausea, vomiting, dysphoria, stomach ache, dyspnea, and even mortality in children. Hydrogen cyanide derived from the amygdalin present in GBSs may cause dizziness, vomiting, contractions, and sleeping disorders. In recent decades, a number of methods have been developed and tested to decrease the contents of individual toxic components. According to literature data, steaming, fermentation, drying, microwave treatment, membrane separation, non-thermal processing, roasting, and boiling the ginkgo seeds decrease toxicity to the degree that allows obtaining a product that is safe to consume while still maintaining most of its active ingredients [12,31]. Most cases of ginkgo seed poisoning resolve without lasting consequences. Many reports describe adverse effects and/or the presence of ginkgotoxin (4′-O-methylpyridoxin, MPN) in patients with ginkgo seed poisoning.

## 7. Regulatory Standards

### 7.1. Valeriana officinalis

Valerian, *Valeriana officinalis*, is a pharmacopeia-listed plant described in the European Pharmacopoeia (Ph. Eur.) [13], British Pharmacopoeia (BP) [14], and American Pharmacopoeia (USP) [15] (European Pharmacopoeia Commission, 2024a, British Pharmacopoeia Commission, 2022a, Czech Republic, 2018, United States Pharmacopeial Convention, 2019a). Monographs on valerian root were also published by the WHO [16], ESCOP [17], EMA [18], and German E Commission [19].

*Valeriana officinalis* plant substances are not mentioned in the Novel Food Status Catalog [20]. The Italian office listed *Valeriana officinalis* (root and vines) as a plant approved for use in dietary supplements [71]. The German office approved the use of valerian root products only as flavor ingredients [72]. Czech regulation approved the use of dry *Valeriana officinalis* root as an ingredient of dietary supplements at a maximum daily dose of 1000 mg [73]. In Belgium and France, the underground parts of valerian are approved for use as dietary supplements, provided that they do not contain valepotriates. Additionally, valerenic acid is monitored in France [74,75]. In the EFSA Compendium, *Valeriana officinalis* root was listed as a negatively evaluated substance or substance subjected to limitations in terms of use in the territory of at least one Member State, for which the Scientific Committee, having analyzed the existing data, was not able to identify the substances that raise concerns [76].

The HMPC report stated that, despite promising results on the use of valerian root extract in children, the data is still too scarce to justify generalized recommendations. Recommendations on the use of valerian root in children below the age of 12 should be supported with specific clinical experience. It is however not necessary to issue special warnings for adolescents above the age of 12, considering the great tolerance of valerian root and its preparations [8].

According to the EMA, valerian preparations cause minor adverse effects. The most commonly reported adverse effects observed during clinical trials included headaches, gastro-intestinal problems, drowsiness, depression, irritability, and nausea. In many studies, adverse effects were not reported [8]. An ESCOP monograph reflects on three clinical trials on the safety of using valerian root (Table 4). The reported adverse effects were determined to be mild to moderate and included headache, a sensation of being dazed in the morning, migraine attacks, gastro-intestinal problems, and accidents [17].

The WHO monograph mentions the following adverse effects of chronic valerian use: headaches, excitability, anxiety, and insomnia. Large doses may cause bradycardia and heart rhythm disturbances, as well as decreased intestinal peristalsis. Doses up to 20-fold higher than the recommended therapeutic doses caused mild symptoms that improved over 24 h. Four cases of liver damage were noted, and they were related to the use of valerian products; however, in all these cases, the patients were taking multi-ingredient plant products; hence, the direct cause is difficult to determine, according to the WHO [84]. The E Commission does not mention adverse effects related to the use of *Valeriana officinalis* root [18]. The available pharmacodynamic studies in animals and people did not confirm the potential interactions of valerian [8]. Moreover, no interactions between the use of valerian preparations and alcohol were noted; nevertheless, it is not recommended to consume alcohol or synthetic sedatives when valerian preparations are used [84]. The minimum daily dose recommended by the EMA monograph is 0.3 g of dry ground plant substance for preparing infusions or 0.3 g of powdered plant substance [19].

Medicinal products with valerian preparations should be used only in adults and adolescents older than 12. Valerian is not recommended during pregnancy and breastfeeding without medical consultation, as information about reproductive toxicity and developmental toxicity is limited. Patients should consult their doctor if symptoms do not improve during the treatment of stress, facilitating sleep, and alleviating nervous tension [8,17].

According to the EMA monograph, *Species sedative*, ground *Valeriana officinalis* L., *radix*, plant substance may be an ingredient of brewing herbs that contain hop cones, *Humulus lupulus* L., *flos*, lavender flower, *Lavandula angustifolia* Mill., *flos*, lemon balm leaves *Melissa officinalis* L., *folium*, and passionflower plant *Passiflora incarnata* L., *herba* [73].

### 7.2. Passiflora incarnata

*Passiflora incarnata* is a pharmacopeia-listed plant described in Ph. Eur. [85] and BP [86]. Monographs on passionflower were published by ESCOP [87] and the German E Commission [68]. The FDA classified passionflower extract (no data on the part of the plant or standardization) as Generally Regarded as Safe (GRAS) [3].

*Passiflora incarnata* is mentioned in the Novel Food Status Catalog [20]. *Passiflora incarnata* (leaf, flower, plant with flowers) was approved in Italy for use in dietary supplements [71]. In Germany, passionflower products were approved for use in foods only in the form of herbs for brewing [72].

According to the quoted EMA report, products with *Passiflora incarnata*, *herba*, may cause drowsiness and impair driving and the use of machines. There are scientific reports of possible interactions with drugs that affect the central nervous system, e.g., lorazepam, and of alcohol’s effects being intensified [9,68].

The ESCOP monograph provides the following information on using passionflower preparations during pregnancy and breastfeeding: it is assumed that the use of dry extracts, liquid extracts, and native infusions of passionflower plant is safe during the 1st and 2nd trimesters and during breastfeeding, after consulting a physician. It is not recommended in the 3rd trimester [68]. A summary report from the EMA does not recommend using passionflower during pregnancy and breastfeeding [9]; a risk for newborns also cannot be excluded [54,87].

### 7.3. Ginkgo biloba

*Ginkgo biloba* is a pharmacopeia-listed raw material. Monographs for the leaf and extract from ginkgo are described in Ph. Eur. [88], BP [89], and USP [90]. Separate monographs for the ginkgo leaves and extract were developed by the E Commission [91]. Ginkgo leaf was also described in the monographs of ESCOP [69], the EMA [88], and the WHO [7]. Plant substance *Ginkgo biloba* was placed in the EFSA Compendium of botanical substances. Parts of plants that may pose potential threats include leaves and seeds (nuclei). Alkylphenols (ginkgolic acids, bilobole, cardanols, cardols, and ginkgols) obtained from leaves, and ginkgotoxin are chemicals of concern. In the comments on adverse or toxic effects that are not known to be related to the identified substance/chemicals of concern, substances present in leaves were mentioned, i.e., sesquiterpene lactones such as bilobalide and diterpene lactones such as ginkgolides, at amounts of 0.06–0.23%. The above-mentioned comments also include seeds, because in Japan, cases of human poisonings were found, including fatalities. The Compendium states that preserved and boiled seeds contain only 1% of the ingredients present in fresh seeds. Roasted seeds also contain ginkgotoxin. The poisoning symptoms include vomiting, seizures, and loss of consciousness [85].

*Ginkgo biloba* leaves are listed in the EFSA Novel Food Status Catalog with a status “Not Novel in Food”. This means that, according to information available to competent authorities of member states, this product was significantly used for consumption by people in the European Union before 15 May 1997. Hence, it is not considered “Novel” as per the regulations on novel foods (EU) 2015/2283, and its market access is not subject to approval prior to marketing as per regulation (EU) 2015/2283 [20]. *Ginkgo biloba* L. leaves are approved for use in dietary supplements in Belgium, France, and the Czech Republic [73,74,75]. French regulations qualify ginkgolic acids, terpene lactones, and flavone glycosides as substances to be monitored. Additionally, France allows the consumption of roasted seeds [75]. Italy approves leaves and buds as dietary supplements [12].

In Germany, the use of *Ginkgo biloba* in food products is limited. The German list places a warning about possible allergic reactions due to the presence of ginkgolic acids. It also indicates critical substances, i.e., lactone diterpenes and ginkgolic acids [72]. The EMA indicates a maximum concentration of summed alkylphenols (e.g., ginkgolic acids, ginkgole, and bilobole) of 5 mg/kg [12]. All referenced regulations exclude the use of ginkgo leaves during pregnancy and breastfeeding, and the Italian office warns against the simultaneous use of anti-clotting and antiplatelet agents [12].

According to ESCOP, there are no limitations concerning the duration of using *Ginkgo biloba* leaf preparations. For dementia, they should be used for at least 12 weeks or more, if health improvement is observed after this time. In patients using a single dose higher than 600 mg of dry ginkgo leaf extract, there were no severe adverse effects [11].

According to the summary report from the EMA, an unequivocal effect of the studied ginkgo leaf extracts on cytochrome P450 (CYP) enzymes was evidenced. The report allows for the interaction of ginkgo extracts with other medicinal products. The results of interaction studies from various experiments suggest that, depending on the administered dose, ginkgo may have both inducing and inhibiting effects on CYP3A4. The literature indicates potential pharmacological interactions between ginkgo and alprazolam, haloperidol, trazodone, omeprazole, aspirin, ibuprofen, nifedipine, warfarin, anti-epileptic medications, antidiabetics, diuretics, and non-steroid anti-inflammatory drugs. However, in another study described in the report, the authors state that the simultaneous administration of *Ginkgo biloba* with aspirin, warfarin, and antipirine showed no pharmacodynamic interactions. However, the combination of ginkgo and cilostazole may increase the risk of hemorrhage. Ginkgo leaf products may decrease the plasma level of Efavirenz (EFV), a medicine used to treat human immunodeficiency virus (HIV) infection, by inducing CYP3A4, an enzyme that metabolizes EFV, which can lead to ineffective therapy [12].

In its report on dry ginkgo extract, the EMA mentions the following adverse effects: bleeding from individual organs (eye, nose, brain, and gastro-intestinal tract), headache, dizziness, mild gastro-intestinal symptoms (such as diarrhea, stomachache, nausea, and vomiting), hypersensitivity reactions (allergic shock), and allergic skin reactions (erythema, edema, pruritus, and rash) [12].

The summary report from the EMA contains a warning about an increased risk of attacks in epileptic patients and patients taking medications that lower the seizure threshold [12].

There is evidence that preparations containing ginkgo may increase the tendency for bleeding. Hence, ginkgo leaf preparations should not be used during pregnancy. The EMA also warns patients with pathologically increased bleeding tendency against the simultaneous use of ginkgo with anti-clotting and antiplatelet agents. The EMA also recommends a prophylactic measure to discontinue ginkgo preparations 3–4 days before a surgical procedure [12].

### 7.4. Extract Standardization and Comparability of Clinical Data

One of the main difficulties in interpreting clinical trials of these three species is the heterogeneity of the preparations used. Herbal extracts are not single substances; their composition depends on the plant material, the solvent, and the extraction process, and this directly affects both efficacy and safety [5].

Ginkgo illustrates the point most clearly. Most of the positive cognitive trials used defined, standardized extracts, above all EGb 761, standardized to 22–27% flavone glycosides; 2.8–3.4% ginkgolides A, B, and C; and 2.6–3.2% bilobalide, with ginkgolic acids restricted to below 5 ppm; however, a smaller number used LI 1370. Trials that used non-characterized ginkgo leaf extract cannot be directly compared with these, because neither the active constituents nor the content of toxicologically relevant ginkgolic acids is known. For valerian, the extract LI 156 was used in the comparison with oxazepam, whereas other trials used aqueous or hydroalcoholic extracts of unstated composition; given that the pharmacologically relevant constituents differ between aqueous and alcoholic extracts, this alone can explain part of the inconsistency between studies. For passionflower, extract characterization is reported even less often, and the plant part and preparation frequently differ between trials.

A recurring limitation across the trials reviewed here is therefore the incomplete reporting of extract identity and standardization. Where the extract is not defined, a positive or negative result cannot be generalized to other products on the market, and pooling such data is not justified. This variability is also the reason why regulatory monographs (Ph. Eur., EMA/HMPC) specify marker content and limits for constituents of concern, and it is a strong argument for requiring full extract characterization in future trials.

## 8. Current State and Perspectives

Despite the popularity of various raw plant materials and their use as medicinal products or dietary supplements, many plants are not well-studied for their mechanisms of activity, toxicity, and clinical effects. Hence, not all indications resulting from traditional applications have been confirmed in research, and the potential of individual plants is not fully harnessed. For plant medications, consumption is controlled by a physician, but with dietary supplements, consumers often believe that herbs are safe, have no adverse effects, and can be used across a broad range of doses. Such an approach may eliminate the positive effects of the plants and their preparations that would be derived from their correct use, as well as threatening health/life. Hence, pharmacological studies on plant-based medicines are arguably necessary to determine their best efficacy in individual conditions, including conditions related to the CNS, and their safety [92,93]. This review summarizes and analyzes the available data and information on the efficacy and safety of *Valeriana officinalis*, *Passiflora incarnata*, and *Ginkgo biloba* in the context of their effects on conditions due to disturbed CNS function, such as ADHD, Parkinson’s disease, migraine, insomnia, anxiety disorders, and dementia, considering the results of clinical trials, regulatory aspects, toxicology, and use limitations (e.g., interactions, contraindications, and use in children and in the elderly). Literature analysis indicated neuromodulatory potential for specific, well-defined indications. The strength of the clinical evidence varies considerably by indication. The most consistent support exists for valerian and passionflower as mild hypnotic and anxiolytic agents, and for standardized ginkgo extracts in mild cognitive impairment and dementia. In contrast, the evidence for ADHD, Parkinson’s disease, and migraine is preliminary, resting on single or small studies and, in some cases, on fixed-combination products, and it does not yet justify clinical recommendations. Noteworthy are the minor adverse effects of the plants and their preparations, as well as beneficial effects observed in clinical trials, i.e., improved quality of sleep, anxiolytic effects, or cerebral circulation improvement. One should also bear in mind the limitations that consumers should be aware of when taking herbal products, as with both dietary supplements and medicinal products. All the mentioned plants should be taken by adults; their intake is not recommended in children younger than 12 (ginkgo can be used by people older than 18). These plants should not be used by pregnant women or lactating women without consulting a doctor. In the case of valerian and passionflower, the warnings also concern the simultaneous use of alcohol, synthetic sedatives, and negative influences on driving or the use of machines. Ginkgo has more contraindications. It is not recommended in people at increased risk of epileptic seizures; those with a pathologically increased bleeding tendency; and those taking medications to lower the seizure threshold, medications influencing blood clotting, or anti-viral agents (HIV). Ginkgo can also influence the metabolism of medications that are eliminated through CYP3A4-, CYP2C9-, or CYP2C19-dependent pathways [70].

*Valeriana officinalis*, *Passiflora incarnata*, and *Ginkgo biloba* are pharmacopeia-listed raw materials; EMA, ESCOP, and WHO monographs have also been published. Preparations of *Valeriana officinalis* are used as medicinal products with well-established effects and traditional applications. *Passiflora incarnata* medicinal products also have the status of medications with traditional application. However, their statuses as dietary supplements and applicable limitations differ between EU countries. They concern both the approved parts of the plant, the preparation form, and the approved dose range or need to monitor specific active or potentially toxic ingredients. *Valeriana officinalis* may be used in dietary supplements only in some countries (e.g., the Czech Republic), while in others (e.g., Germany), its use is limited to flavoring functions. In France and Belgium, there are additionally strict limitations on the content of valepotriates, and in France, valerenic acid is monitored. The use of *Passiflora incarnata* in dietary supplements is also limited, e.g., in Italy to chosen parts of the plant (leaf, flower, plant with flowers), and in Germany only as a raw material for infusion. *Ginkgo biloba* has broader applications in dietary supplements—the use of the leaves is approved in Belgium, France, and the Czech Republic, while the use of the leaves and buds is approved in Italy; however, there are also significant toxicological concerns. In France and Germany, regulations are in force on the maximum contents of ginkgolic acids, terpene lactones, and flavone glycosides. Moreover, the EFSA and toxicological reports highlight potential threats related to the presence of ginkgotoxin, alkylphenols, and other toxic ingredients, especially in fresh seeds. Even small amounts of these substances can cause acute poisoning, which justifies limitations on their use or mandates processing before consumption [29].

Without a doubt, the raw plant materials presented in this article influence the CNS, with effects that are potentially therapeutic for anxiety disorders, insomnia, nervous distress, memory disturbances, concentration disorders, ADHD, and Parkinson’s disease. Despite the promising results of studies, the use of these plants in clinical practice requires further research, especially randomized controlled trials with the participation of larger patient groups. Potential interactions with conventional medications and differences in the quality and standardization of plant preparations need to be considered. Hence, complex summaries extending beyond the results of clinical trials are needed.

## 9. Conclusions

*Valeriana officinalis*, *Passiflora incarnata*, and *Ginkgo biloba* show real but uneven promise in CNS-related conditions. The evidence is most consistent for valerian and passionflower as mild hypnotic and anxiolytic agents and for standardized ginkgo extracts in mild cognitive impairment and dementia, whereas their use in ADHD, Parkinson’s disease, and migraine remains preliminary. Across all three, safe use depends on awareness of contraindications, interactions, and the variability of the available preparations. Realizing their full therapeutic potential will require larger, well-designed randomized controlled trials with properly characterized, standardized extracts.

## Figures and Tables

**Table 1:** Main phytochemical constituents of *Valeriana officinalis*, *Passiflora incarnata*, and *Ginkgo biloba*.

Species/Plant Part	Main Constituent Groups	Key Marker Compounds	Typical Content
*Valeriana officinalis* Root and rhizome	Iridoids (valepotriates), sesquiterpenic acids, essential oil, lignans, flavonoids	Valtrate, acetylvaltrate, dihydrovaltrate, valerenic acid, hydroxyvalerenic acid	Valepotriates 0.5–2%; valerenic acid ≥ 0.17% (Ph. Eur.)
*Passiflora incarnata* Aerial parts (herba)	Flavonoids (C-glycosyl flavones), maltol, trace harmala alkaloids, phytosterols	Vitexin, isovitexin, orientin, isoorientin, schaftoside	Total flavonoids ≥ 1.5% expressed as vitexin (Ph. Eur)
*Ginkgo biloba* Leaf (standardized extract)	Flavonol glycosides, terpene lactones (ginkgolides, bilobalide), proanthocyanidins; alkylphenols (ginkgolic acids)	Quercetin, kaempferol, and isorhamnetin glycosides, ginkgolides A, B, C, bilobalide	Flavone glycosides 22–27%, ginkgolides A–C 2.8–3.4%, bilobalide 2.6–3.2%, ginkgolic acids ≤ 5 ppm (Ph. Eur.)

**Table 3:** Comparative summary of *Valeriana officinalis*, *Passiflora incarnata*, and *Ginkgo biloba*: principal CNS indications, strength of clinical evidence, standardized extracts, main safety concerns, and EU regulatory status.

Feature	*Valeriana officinalis*	*Passiflora incarnata*	*Ginkgo biloba*
Plant part used	Root and rhizome	Aerial parts (herba)	Leaf (standardized extract), seeds toxic, not used therapeutically
Best-supported CNS indication	Insomnia, mild sleep disturbance	Anxiety (incl. preoperative), mild sedation	Mild cognitive impairment and dementia
Strength of clinical evidence	Moderate: several small RCTs and active-comparator trials (e.g., vs. oxazepam)	Moderate for anxiety, limited for insomnia: several small, short RCTs	Strongest for cognition (multi-center RCTs, standardized extract), GuidAge negative for prevention
Evidence for other indications	ADHD, OCD: preliminary	ADHD, opiate withdrawal: preliminary	ADHD not superior to methylphenidate, Parkinson’s, migraine: preliminary
Standardized extract(s) in trials	LI 156, other aqueous/hydroalcoholic extracts of unstated composition	Rarely characterized, plant part and preparation vary	EGb 761, LI 1370, several trials used non-characterized extract
Key constituents of concern	Valepotriates (unstable, low bioavailability)	Harman alkaloids (isolated cardiac/GI effects)	Ginkgolic acids, alkylphenols (≤5 ppm), ginkgotoxin, cyanogens in seeds
Main interactions/warnings	Potentiates CNS depressants and alcohol; caution when driving	Sedative/alcohol potentiation, possible lorazepam interaction, driving	Bleeding risk, CYP3A4/CYP2C19, caution with anticoagulants, antiplatelets, seizure-threshold drugs, stop 3–4 days before surgery
Use in children	Not < 12 years	Not established < 12 years	Not < 18 years
Pregnancy/lactation	Not without medical advice	Not in 3rd trimester, caution otherwise	Contraindicated
EU status (medicinal product)	Well-established use and traditional use	Traditional use	Well-established use and traditional use (leaf extract)
Status as supplement ingredient (examples)	Czech Republic: ≤1000 mg/day; Germany: flavoring only; Belgium/France: valepotriate limits; France: valerenic acid monitored	Italy: (leaf, flower, flowering plant), Germany: only as infusion herb	Belgium, France, Czech Republic: Leaf, Italy: leaf + buds, France: ginkgolic acids/terpene lactones/flavone glycosides monitored, Germany: allergy warning

Abbreviations: GI—gastro-intestinal; OCD—obsessive–compulsive disorder; RCT—randomized controlled trial.

**Table 4:** Summary of available toxicological data on *Valeriana officinalis*, *Passiflora incarnata*, and *Ginkgo biloba*.

Medicinal Plant	Study Model/Experimental Organisms	Type of Study	Substance/Extract	Dose	Duration	Toxicological Effect	Ref.
*Passiflora incarnata*	Mice, rats	Acute	Dry aqueous ethanol extract	LD_50_ p.o. > 15 g/kg (mice, rats)	NA	LD_50_ p.o. > 15 g/kg (mice, rats)	[9]
	Rats, mice	Acute	Dry aqueous ethanol extract	LD_50_ i.p.: 3510 mg/kg (rats), 3140 mg/kg (mice)	NA	LD_50_ i.p.: 3510 mg/kg (rats), 3140 mg/kg (mice)	
	Rats, mice	Acute	Dry aqueous ethanol extract	LD_50_ s.c.: >10 g/kg (rats), 8300 mg/kg (mice)	NA	LD_50_ s.c.: >10 g/kg (rats), 8300 mg/kg (mice)	
	Mice	Acute	Maltol	LD_50_ s.c.: 820 mg/kg	NA	LD_50_ s.c.: 820 mg/kg	
	Mice	Acute	Ethyl maltol	LD_50_ i.p.: 910 mg/kg	NA	LD_50_ i.p.: 910 mg/kg	
	Rats	Chronic	Dry aqueous ethanol extract (no DER)	10 mL/kg p.o. (5 g/kg dry mass)	21 days	No effect on body weight, temperature, motor coordination	[68]
	Rats	Sub-chronic	Dry aqueous ethanol extract	600 mg/kg p.o.	4 weeks	No toxic effects	[9,68]
	Mice	Sub-chronic	Leaf and fruit extract (60% ethanol, 8:2)	500 mg/kg i.p.	5 days	No changes in body weight, food and water intake; no histological lesions	[68]
	Aspergillus nidulans D-30	Genotoxicity	Liquid extract (16.2% of dry mass)	1.30 mg/mL	NA	No mutagenic effects (somatic segregation assay)	
	Mice	Acute	Lyophilized aqueous extract	>900 mg/kg i.p.	48 h	No acute toxicity	[77]
	Mice	Acute	Leaf extract (petroleum ether, methanol)	50–1600 mg/kg p.o.	7 days	No mortality	[78]
*Ginkgo biloba*	Mice	Acute	EGb 761	LD_50_ p.o. 7.73 g/kg; LD_50_ i.v.: 1.1 g/kg; LD_50_ i.p.: 1.9 g/kg		LD_50_ p.o. 7.73 g/kg; LD_50_ i.v.: 1.1 g/kg; LD_50_ i.p.: 1.9 g/kg	[12]
	Rats	Acute	EGb 761	LD_50_ i.v.: 1.1 g/kg; LD_50_ i.p.: 2.1 g/kg		Up to a dose of 10 g/kg p.o., the extract caused no fatalities, and administration of higher doses was not possible	
	Rats, mice, dogs	Chronic	EGb 761	100–1600 mg/kg p.o.	27 weeks (rodents), 26 weeks (dogs)	No organ damage, no disturbances in renal and hepatic functions	
	Rats		MNP	50 mg/kg bw p.o	15 days	Brain and heart damage	[79]
	Mice	Effect on oocyte maturation and further development before and after implantation in vitro and in vivo	Ginkgolide B (GKB)	1–6 µM GKB in vitro, 3–6 µM GKB in vitro	4 days	Decreased rate of oocyte maturation and in vitro fertilization, and damage at the early stage of embryo development, especially inhibition of development from blastocyst stage	[80]
*Valeriana officinalis*	Mice	Acute/neurotoxicity	Valeric acid	50–400 mg/kg i.p.	—	Neurotoxic effects (ataxia, seizures, mortality at 400 mg/kg)	[81]
	Rats	Chronic	Ethanol extract	300–600 mg/kg p.o.	30 days	No organ lesions and no changes in blood parameters	[82]
	Rats	Acute, subchronic	Unspecified root extract	0.31–18.6 g/kg p.o. (single); 3.1 g/kg p.o. daily	1–28 days	No changes in liver enzymes, microcirculation, and histology	[83]

NA—Not Available.

## Data Availability

No new data were created or analyzed in this study. Data sharing is not applicable to this article.
